# Enhancing Security and Privacy in Healthcare Systems Using a Lightweight RFID Protocol

**DOI:** 10.3390/s23125518

**Published:** 2023-06-12

**Authors:** Muhammad Ayaz Khan, Subhan Ullah, Tahir Ahmad, Khwaja Jawad, Attaullah Buriro

**Affiliations:** 1 Department of Computer Science, Air University, Islamabad 44000, Pakistan; ayaz.khan@mail.au.edu.pk; 2 Faculty of Computer Science, National University of Computer and Emerging Sciences (NUCES-FAST), Islamabad 44000, Pakistan; subhan.ullah@nu.edu.pk; 3 Center for Cybersecurity, Brunno Kessler Foundation, 38123 Trento, Italy; 4 Department of Computer Science, Iqra National University, Swat 19200, Pakistan; khwajajawad@inuswat.edu.pk; 5 Faculty of Engineering, Free University Bozen-Bolzano, 39100 Bolzano, Italy

**Keywords:** RFID protocol, Internet of Healthcare Things, RFID authentication, IoT security

## Abstract

Exploiting Radio Frequency Identification (RFID) technology in healthcare systems has become a common practice, as it ensures better patient care and safety. However, these systems are prone to security vulnerabilities that can jeopardize patient privacy and the secure management of patient credentials. This paper aims to advance state-of-the-art approaches by developing more secure and private RFID-based healthcare systems. More specifically, we propose a lightweight RFID protocol that safeguards patients’ privacy in the Internet of Healthcare Things (IoHT) domain by utilizing pseudonyms instead of real IDs, thereby ensuring secure communication between tags and readers. The proposed protocol has undergone rigorous testing and has been proven to be secure against various security attacks. This article provides a comprehensive overview of how RFID technology is used in healthcare systems and benchmarks the challenges faced by these systems. Then, it reviews the existing RFID authentication protocols proposed for IoT-based healthcare systems in terms of their strengths, challenges, and limitations. To overcome the limitations of existing approaches, we proposed a protocol that addresses the anonymity and traceability issues in existing schemes. Furthermore, we demonstrated that our proposed protocol had a lower computational cost than existing protocols and ensured better security. Finally, our proposed lightweight RFID protocol ensured strong security against known attacks and protected patient privacy using pseudonyms instead of real IDs.

## 1. Introduction

The Internet of Things (IoT) is a rapidly growing communication paradigm in various fields, including healthcare [1,2,3,4,5]. It involves connecting different physical objects through the internet, thereby allowing automated events and activities to occur. Integrating physical infrastructure with information technology has led to several IoT domains, including healthcare, which has revolutionized the healthcare industry by providing the real-time monitoring of patients and medical equipment [2,6,7].

Despite the numerous advantages of the IoT in the healthcare industry, security and privacy concerns are associated with it. Sensitive personal information is often transferred over an unreliable communication network, leaving it vulnerable to attacks. Moreover, RFID platforms offer a promising solution, but security and privacy concerns remain top priorities. In particular, an attacker could capture, alter, or manipulate patient data, thereby potentially harming patients and medical devices. These concerns are amplified when patients receive IoT facilities over a shared network, thus resulting in more data protection, authenticity, and accessibility-related issues. Therefore, there is a need for a trustworthy and secure RFID authentication system for the IoT health industry to address these concerns.

**Figure 1:**
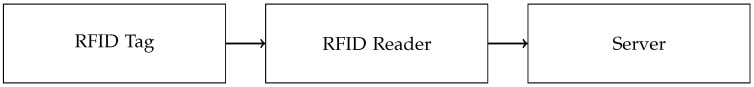
Architecture of an RFID System.

Radio-Frequency Identification (RFID) systems have gained widespread attention in the healthcare industry for over a decade, wherein they allow for the easy tracking of patients, hospital supplies, medicine, and medical equipment. The architecture of an RFID system (as shown in Figure 1) comprises three main components: reader, back-end server, and tag. The reader gathers data from the tag and updates or verifies it via the back-end server. The tag contains hardware for processing information, an antenna for sending and receiving signals from the reader, and a microchip that stores sensitive data, such as passwords and authentication protocols. The server is considered an authentic entity that stores all the identities of tags and other important information, which helps to establish the reader and tag’s mutual authentication. RFID sensors, connected via an armband, can store patient information, which a doctor can quickly retrieve using a reader. However, the tracking capabilities of RFID systems raise security and privacy concerns. To address these concerns, authentication is a core security measure for recognizing tags, as the reader must know which tag to track [6,8,9].

The main contribution of this work is the proposal of a new lightweight authentication approach for RFID-based systems in the IoT-based healthcare domain. While previous research [10,11,12,13,14] has tried to develop secure and resilient RFID authentication schemes, vulnerabilities still exist. Therefore, this paper addresses these limitations by introducing an improved authentication scheme that offers enhanced protection compared to existing approaches.

Performance evaluation was conducted to assess the efficiency and effectiveness of the proposed protocol compared to state-of-the-art approaches. The evaluation included a computational cost comparison, which measured the computational resources required by the protocol. By benchmarking against existing protocols, the performance evaluation demonstrated the superiority of the proposed protocol in terms of computational efficiency.

For the security analysis, formal verification techniques were employed to ensure the robustness of the proposed protocol against potential security threats. Specifically, the protocol underwent scrutiny using ProVerif, which is a widely recognized formal verification tool for security protocol analysis. Queries were formulated to assess various security properties, such as resistance against event injection and protection against attackers. The responses from ProVerif validated that the proposed protocol satisfied the specified security requirements and could withstand potential security attacks.

In addition to the formal verification technique using ProVerif, this study employed BAN logic for conducting a comprehensive security analysis of the proposed lightweight RFID protocol. BAN logic is a formal modelling and analysis technique designed for security protocols. It enables the specification of security properties and the verification of protocol behaviour against those properties. The proposed protocol was thoroughly examined by leveraging BAN logic to assess its security properties and ensure its resistance against potential attacks. The analysis considered various security aspects, such as tag anonymity, replay attack resistance, synchronization attack resistance, forward secrecy, mutual authentication, anti-DoS attacks, impersonation attacks, insider attacks, and other relevant security concerns.

Similarly, the informal security analysis compared the proposed scheme with existing protocols, thereby revealing its superiority in meeting all the listed security criteria. The proposed scheme outperformed other protocols, thus demonstrating its effectiveness in ensuring tag anonymity, protection against attacks, mutual authentication, and more.

The rigorous security analysis and comprehensive performance evaluation ensured that the proposed lightweight RFID protocol provided enhanced security and privacy, as well as offered efficient and effective performance. This holistic approach guaranteed the protocol’s suitability for deployment in real-world healthcare systems, where security and performance are critical factors.

In summary, this paper aims to enhance the security and privacy of healthcare systems by proposing a lightweight RFID protocol. The proposed protocol addresses existing schemes’ anonymity and traceability issues by utilizing pseudonyms instead of real IDs and ensuring secure communication between tags and readers. The protocol has undergone rigorous testing and has been proven to be secure against various security attacks. Furthermore, the paper provides an overview of how RFID technology is used in healthcare systems and highlights the challenges faced by these systems. It reviews existing RFID authentication protocols proposed for IoT-based healthcare systems, wherein it discusses their strengths, challenges, and limitations. To overcome the limitations of existing approaches, the proposed protocol was introduced, which provided better security and had a lower computational cost than existing protocols. It ensured security against known attacks and protected patient privacy by utilizing pseudonyms. By introducing this novel lightweight RFID protocol and conducting a thorough evaluation using formal verification techniques, this study contributes to the advancement of secure RFID protocols for IoT-based healthcare systems. The proposed protocol aims to address the security and privacy concerns associated with RFID-based healthcare systems, thereby ultimately ensuring better patient care and safety.

## 2. Related Work

This section reviews the existing approaches related to the authentication and privacy of patients in the Internet of Healthcare Things (IoHT). These approaches mostly investigated RFID-based authentication solutions using ECC, inbuilt ECC ID verifiers, PUF, a one-way hash with a straightforward bitwise exclusive-OR function, and URASP for RFID. These approaches partially overcome the privacy, authentication, and integrity issues from impersonation, loss, replay, and de-synchronization attacks. This section further discusses the strengths, challenges, and limitations of the existing approaches and identifies the gap in the literature. The gap analysis leads the discussion to our proposed RFID protocol, which safeguards patients’ privacy in the IoHT domain by utilizing pseudonyms instead of real IDs, thereby ensuring secure communication between tags and readers. In the existing approaches, Kaul et al. [15] offered a privacy-preserving and efficient authentication protocol (RFID) consisting of initialization, authentication, and updating phases for healthcare systems. The protocol intended to secure communication between RFID tags and readers with patient privacy using pseudonyms instead of real IDs, where the tag would update a pseudonym upon each successful authentication operation between a tag and server. However, the server would store it until synchronization with the new one. They also used a one-way hash function and bitwise XOR operation. Chou et al. [16] proposed an RFID-based authentication using ECC to address security issues such as impersonation, de-synchronization attacks, and tag tracking. They claim their protocol is secure against known threats, including Man-in-the-Middle (MITM) and replay attacks.

However, Zhang et al. [16] found the Chou et al. [16] scheme to be unsafe against impersonation attacks, and they proved that the scheme had no forward security. Liao et al. [17] proposed a secured RFID system with an inbuilt ECC ID verifier protocol for the medical environment. Their proposed protocol provided various safety features but was insecure if an adversary revealed the secret key of a tag [18]. The scheme had no resistance against impersonation attacks. Moreover, the Liao et al. [17] scheme had no resistance against location privacy, tag cloning, and tag masquerades, as revealed by Peeters et al. [19].

Zhao et al. [18] also presented a secure RFID system with ECC. However, Farash et al. [20] realized that the proposed scheme did not preserve any forward secrecy in the system, and, therefore, they offered a proven ECC-based secure RFID system for healthcare.

Similarly, Srivastava et al. [21] proposed an RFID-based tag of a mutual authentication protocol for a healthcare system. The protocol used a synchronized shared secret, a one-way hash function, and a straightforward bitwise exclusive-OR function. Their approach resisted well-known security threats, including de-synchronization, replay, traceability, and forgery attacks. However, Li et al. [22] revealed in the same year that the Srivastava et al. technique exposed a severe security flaw. An attacker can use a stolen RFID reader to interact with the medical server containing the sensitive data of the tag-based devices. The technique also lacks mutual authentication and is vulnerable to attacks using stolen or lost readers.

Jin et al. [23] also proposed an RFID system to improve patient safety in medication environments. Their scheme used ECC to attain the necessary safety features and resistance for several known security assaults such as Denial-of-Service (DoS), replay, tag impersonation, location tracking, cloning, server spoofing, de-synchronization, and MITM attacks. However, Pokala et al. [24] pointed out that the Jin et al. [23] scheme did not maintain the attribute of tag identity and was prone to impersonation attacks of tags. To address these security flaws and improve the effectiveness of RFID systems, Li et al. [22] proposed an enhancement to the approach suggested by Srivastava et al. [21]. The Li et al. [22] protocol utilized reader-specific identification, reader-specific secret value, bitwise exclusive OR, and lightweight hashing to accomplish mutual authentication while also providing resistance to reader theft or loss, replay, and de-synchronization attacks.

Zhou et al. [25] proclaimed that the Li et al. [22] scheme was not applicable in a mobile RFID context due to the lack of a secure communication channel. As a result, the Li et al. [22] scheme has data integrity issues in a mobile RFID context and is susceptible to de-synchronization, replay, and traceability attacks. To overcome these security issues, Safkhani et al. [26] proposed a novel cryptanalysis of an authentication scheme based on RFID that was suggested by Zheng et al. [27] for mobile devices. They emphasized that their proposed scheme could resist impersonation, replay, and de-synchronization attacks. They also suggested a new protocol that would be safe from other potential attacks.

Chen et al. [28] cryptanalyzed two RFID authentication protocols proposed by Fan et al. [14] and Benssalah et al. [7]. They demonstrated their protocols as being susceptible to tracking, reader, and tag impersonation attacks. Eventually, they suggested an improved RFID-based protocol called TMIS.

Shariq et al. [29] proposed a permutation-based ultralightweight validation mechanism named URASP for RFID. The protocol performs left circular rotation Rot (.,.), bitwise XOR, and permutation (Per(.,.)) processes on passive RFID tags. In addition to privacy protection and untraceability of tagging under Weis and Juel’s privacy model, the protocol can resist various security attacks. They used the Scyther tool and BAN logic to verify the scheme.

Also, Xiao et al. [30] proposed an access control lightweight authentication scheme for TMIS. The protocol can establish secure authentication based on physical unclonable function (PUF)- and ECC-based approaches among the server and tag. The information generated by the PUF overcomes the algorithm cost and prevents data leaks. The ProVerif tool demonstrated that the scheme resists significant threats. Chen et al. [10] proposed an ECC-based RFID authentication scheme and employed power exponentiation that achieved partial security, which makes it suitable for healthcare scenarios. Bilal et al. [11] performed the security analysis of a genetic algorithm called Gossamer protocol that also employed power exaponentiation by launching various attacks, e.g., DoS, exhaustive memory and processing, replay, and IDS collision attacks. They used ROTbits for confusion and MIXbits function for diffusion for cheaper operations and implementations. However, their scheme had weaknesses in the implementation and design. Based on the Gossamer protocol, they proposed an ultra-lightweight protocol and showed its suitability for low-cost RFID tags.

Xie et al. [13] used a VPN to ensure the secure communication of a cloud-based RFID for the authentication of tag preservation, reader privacy, and security of the database owners. They used a hash operation and prevented a location tracking attack. However, their scheme had a computational overhead and needed more operations for symmetric decryption on the reader side due to the exchange of a large amount of data between the reader and the cloud. Sarah et al. [12] prevented the attacks and minimized tag overhead by proposing a lightweight protocol. They also used hash operations and protected privacy of the tags, used permutation and rotation instead of hashing for data encryption, and reduced the computational cost. They proposed timestamps for the updated information and freshness of the message that avoided de-synchronization attacks and protected tag privacy.

In the scheme suggested by Fan et al. [14], they claimed resistance to all known attacks. However, we found that Fan et al. [14] had several weaknesses, as the adversary intercepts the value of $N_{R}$, which conveys over the public channel from the reader to the tag. The reader encrypts TID with $N_{R}$ and sends the encrypted value $\left(TID⊕N_{R}\right)$ to the tag over a public channel. The adversary intercepts this encrypted value and performs an XOR operation to obtain TID. The adversary calculates the original identity of the tag, TID, based on the intercepted and encrypted value sent by the reader to the tag over the public channel. The exposure of TID can lead to the issues of tag anonymity and tag traceability. This scheme uses displacement operation, which costs more than the other operations. Overall, this work reviews the existing RFID authentication protocol and its strengths, challenges, and limitations in IoT-based healthcare systems. It also highlights the importance of secure and private healthcare systems using RFID technology and provides insights into the existing solutions and their weaknesses. As discussed above, most of the literature offers privacy-preserving and efficient authentication approaches. Some are addressing impersonation, de-synchronization, and tag-tracking attacks. However, these approaches still have challenges that include forward secrecy, the revelation of the secret key of a tag, and the lack of mutual authentication, where the attacker can use a stolen RFID reader to interact with the medical server. Maintaining the tag identity is also a challenge, which is prone to the impersonation attacks of a tag. To address these security flaws and to improve the effectiveness of RFID systems, a reader-specific identification has been utilized and accomplished mutual authentication while providing resistance to reader theft or loss, replay, and de-synchronization attacks. The lack of a secure channel also still results in data integrity issues, e.g., de-synchronization, replay attacks, and traceability attacks in mobile RFID scenarios. Using PUF- and ECC-based approaches can overcome the algorithmic cost and prevention of data leaks. However, the computational overhead and the interception of the encrypted identity value sent by the reader to the tag over a public channel may lead to an issue of tag anonymity and tag traceability.

Our proposed scheme differs from the state-of-the-art approaches, as it employs lightweight operations and requires fewer computing resources. In this paper, we proposed a lightweight RFID protocol that addresses the anonymity and traceability issues found in a system of Fan et al. [14]. Our scheme uses a combination of symmetric key encryption and hash functions to protect patient privacy while ensuring secure communication between tags and readers. Overall, the review of the literature highlights the importance of secure and private healthcare systems using RFID technology and provides insights into existing solutions and their limitations. The proposed scheme is defenceless against stolen verifier attacks and insider impersonation attacks. The server sends $N_{R}$ and $N_{T}$ to the reader over a public channel without encryption of the reader, which sends $N_{S}$ to the tag. This vulnerability can be exploited to launch impersonation attacks. After an impersonation attack, the opponent can calculate a new session key, which makes the scheme vulnerable to session-key attacks.

## 3. Proposed Lightweight RFID Protocol

**Figure 2:**
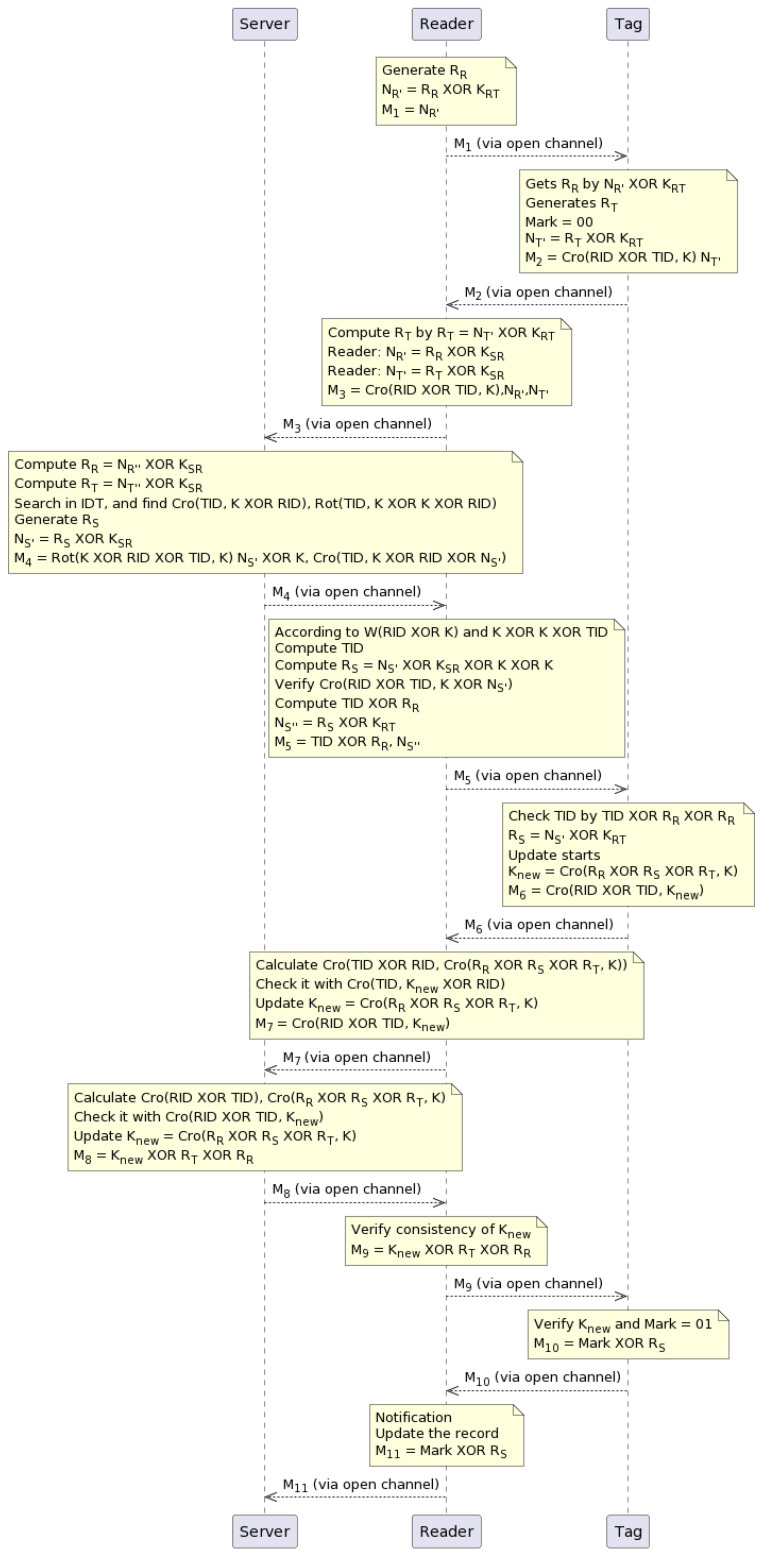
Proposed lightweight authentication scheme.

The proposed scheme is shown in Figure 2, and the steps are explained below. The notations are shown in Table 1.

**Step 1:**  The scheme involves the reader and tag exchanging random numbers. The $R_{R}$ is a random number generated by a reader, and it is encrypted with a preshared key $K_{SR}$ between the reader and tag. The resulting value $N_{R}^{{}^{\prime }}=R_{R}⊕K_{SR}$ is stored by the reader in $M_{1}$, which is a message sent through a public channel to the tag.
**Step 2:**  The tag decrypts the random number by computing $R_{R}=N_{R}^{{}^{\prime }}⊕K_{RT}$, where $K_{RT}$ is a preshared key among the tag and reader. The tag generates its random number $R_{T}$ and sets a mark value of 00, thus indicating the start of the session. The tag then encrypts its random number with $K_{RT}$ and stores the result in $N_{R}^{{}^{\prime }}$ as $N_{R}^{{}^{\prime }}=R_{T}⊕K_{RT}$. The tag also calculates Cro(RID⊕TID,K) and stores it in $M_{2}$, which is sent to the reader through a public channel.
**Step 3:**  The reader decrypts the tag’s random number by computing $R_{T}=N_{T}^{{}^{\prime }}⊕K_{RT}$, where $N_{T}^{{}^{\prime }}$ is the value received in $M_{2}$. The reader then encrypts the tag’s nonce and the reader’s nonce using a preshared key $K_{SR}$ between the server and reader. This results in $N_{R}^{{}^{\prime \prime }}=R_{R}⊕K_{SR}$ and $N_{T}^{{}^{\prime \prime }}=R_{T}⊕K_{SR}$ (the double primes indicate the second encryption). The reader then calculates Cro(RID⊕TID,K) and stores it along with $N_{T}^{{}^{\prime \prime }}$ and $N_{R}^{{}^{\prime \prime }}$ in $M_{3}$, which is sent directly to the server.
**Step 4:**  The server attains the random numbers of the reader and tag by decrypting them with $K_{SR}$ as $R_{R}=N_{R}^{{}^{\prime \prime }}⊕K_{SR}$ and $R_{T}=N_{T}^{{}^{\prime \prime }}⊕K_{SR}$, respectively. The server searches the ID table IDT for the index corresponding to the value received in $M_{3}$, which is Cro(TID⊕RID,K). The protocol stops if the index value does not match an index in IDT. If the index value matches an index in IDT, a $R_{S}$ random number is produced by the server, which then encrypts it with $K_{SR}$ and stores the result in $N_{S}^{{}^{\prime }}=R_{S}⊕K_{SR}$. The server then calculates $Cro(RID⊕TID,N_{S}^{{}^{\prime }}⊕k)$, Rot(K⊕TID,RID⊕k), and $k⊕N_{S}^{{}^{\prime }}$ and stores all three values in $M_{4}$, which is sent to the reader through a public channel.
**Step 5:**  The reader checks the TID and obtains $R_{S}$ as follows. First, it computes the hamming weight of K⊕TID, which is denoted by W(TID⊕K). Then, it computes K⊕K⊕TID. Using these values, it obtains TID and $R_{S}$ as $TID=Cro(TID⊕RID,K⊕N_{S}^{{}^{\prime }})$ and $R_{S}=N_{S}^{{}^{\prime }}⊕KST⊕K⊕K$, respectively. The reader then compares the received value $Cro(TID⊕RID,K⊕N_{S}^{{}^{\prime }})$ with the calculated value to verify. If they match, it stores $TID⊕R_{R}$ and $N_{S}^{{}^{\prime \prime }}=R_{S}K_{RT}$ in $M_{5}$ and forwards $M_{5}$ to the tag through a public channel.
**Step 6:**  The tag first obtains a random number $R_{S}=N_{S}^{{}^{\prime \prime }}⊕K_{KRT}$. Then, it performs an XOR operation between the TID and the previously received $R_{R}$, which is denoted as $TID⊕R_{R}$. Next, it checks if $TID=TID⊕R_{R}⊕R_{R}$. After that, it updates the session number *K* by acquiring three random numbers: $R_{S},R_{R},$ and $R_{T}$. Specifically, *K* is replaced with $K_{new}$, where $K_{new}=Cro\left(N_{R}⊕N_{R}⊕N_{T},K\right)$. Remember that *K* is the default value mutually exchanged by the reader, tag, and server in the first session. Before initiating the next phase, the tag stores $Cro(TID,K_{new}⊕RID)$ in $M_{6}$ and is shared with the reader.
**Step 7:**  The *K* in the server and reader is updated. Since some of the parameters are already calculated and present in the reader and server, such as $RID,TID,R_{S},R_{R},R_{T},$ and *K*, they take advantage of this fact and execute $Cro(RID⊕TID,Cro\left(R_{S}⊕R_{R}⊕R_{T},K\right))$ to obtain $K_{new}$. They then compare it with the $K_{new}$ received from the tag, which is denoted as M7= $Cro(RID⊕TID,K_{new})$. If they match, the reader updates $K_{new}=Cro\left(R_{S}⊕R_{R}⊕R_{T},K\right)$. After this step, some verification operations are performed for the consistency of $K_{new}$ in the tag, reader, and server. Finally, the reader shares M7 with the server.
**Step 8:**  The server calculates Cro(RID⊕TID), and $Cro(R_{R}⊕R_{S}⊕R_{T},K)$ and checks them with $Cro(RID⊕TID,K_{new})$; after that, it updates $K_{new}=Cro\left(R_{R}⊕R_{S}⊕R_{T},K\right)$ and stores $K_{new}⊕R_{T}⊕R_{R}$ in M8. The server sends M8 to the reader via an insecure channel.
**Step 9:**  The reader verifies the consistency of $K_{new}$ and calculates $XORsK_{new}$, $R_{T}$, and $N_{R}$; it then stores them in $M_{9}$ as $M_{9}=K_{new}⊕R_{T}⊕N_{R}$. The reader also sends them to the tag, but it stores them within $M_{9}$ before sending them to the tag. Thus, $M_{9}$ is sent to the tag through a public channel.
**Step 10:**  In addition, both the reader and tag perform the same operations to confirm $K_{new}$ by obtaining it with the help of the operation $\left(K_{new}⊕R_{T}⊕R_{R}\right)⊕R_{T}⊕R_{R}$, and they validate it against the previous value $K_{new}$ that was calculated before. If the verification process does not encounter any problems and is smooth, the Mark value is set to 01, thereby indicating that the synchronization regarding *K* is completed.
**Step 11:**  The reader receives a notification from the tag to update the record. The reader stores mark value XOR with $R_{s}$ in M11; it then forwards Mark to the server, which means the value is 01 at the server side. A new record $\{Cro\left(RID⊕TID,K_{new}\right)$, $Rot(K_{new}⊕TID,K_{new}⊕RID)\}$ is produced and added to the index table IDT. The tag then sets the Mark value to 10 after receiving a notification that the data has finished updating. The proposed authentication protocol is completed.

## 4. Computation Cost Comparison

This section analyzes the protocols’ computational costs and highlights the proposed scheme’s advantages. Table 2 allows us to assess the efficiency of the proposed scheme in relation to existing protocols.

The Kaul et al. [15] RFID scheme has three phases, i.e., initialization, authentication, and updating. These phases perform a PRNG operation for pseudonyms, along with one-way hash functions and bitwise XOR (⊕) operations.

The Chien Protocol [10] employs operations such as XOR (⊕), power exponentiation (∧), cascading operation (||), and displacement operation (Rot). These operations are computationally expensive, especially exponentiation and cascading. The high computational cost of these operations may impact the protocol’s performance, thereby making it less efficient in resource-constrained environments.

The Gossamer Protocol [11] also utilizes XOR (⊕), power exponentiation (∧), and displacement operation (Rot). However, it performs a double displacement operation (Rot${}^{2}$), thereby increasing computational complexity. As a result, the Gossamer Protocol may be more resource-intensive than other schemes.

The Xie Protocol [13] focuses on lightweight operations such as XOR (⊕), cascading operation (||), and hash operation. While these operations have a relatively lower computational cost, the absence of power exponentiation in the protocol limits its overall security and efficiency.

The Sarah Protocol [12] employs a combination of XOR (⊕), power exponentiation (∧), cascading operation (||), and hash operation. Although it offers a comprehensive set of operations, the protocol incurs a higher computational cost due to the involvement of power exponentiation and cascading.

The Fan Protocol [14] utilizes XOR (⊕), cascading operation (||), cross operation (Cro), and displacement operation (Rot). Including cross and displacement operations increases the computational complexity of the protocol. These operations may pose a challenge regarding computational efficiency, especially in resource-constrained environments.

In contrast, the proposed scheme focuses on lightweight operations, primarily XOR (⊕) and a cross operation (Cro). These operations have a lower computational cost than exponentiation, cascading, and displacement operations. By reducing the complexity of operations, the proposed scheme achieves better computational efficiency while maintaining an acceptable level of security. This makes it well-suited for IoT-based healthcare systems, which are often operating in resource-constrained environments.

Overall, the proposed scheme demonstrates a notable advantage in terms of computation cost compared to existing protocols. By utilizing lightweight operations, it minimizes the computational burden without compromising the security requirements. The reduced computational cost translates into improved efficiency, thereby making the proposed scheme a promising choice for secure RFID authentication in healthcare IoT systems.

## 5. Security Analysis

Formal security analysis of the designed scheme was conducted (using ProVerif) and examined informally (BAN logic).

### 5.1. Automated ProVerif Security Proof

ProVerif is a software tool that automates and aids in testing essential security aspects such as authentication, accessibility, and anonymity. Three entities are defined in the proposed lightweight scheme—server, tag, and reader—so we need to define four queries—three for each entity and the last for an attacker—to indicate that the secret key is secure and the attacker will not be intercepted.

The description of each query is as follows.

- Query 1 tests the event injection for the server. It checks if the ProVerif response confirms that the connection on the server is successfully opened and closed. The query result indicates that the event injection from end_S(IDS[]) to start_S(IDS[]) is true, meaning that the server’s communication channel is functioning correctly.
- Query 2 tests the event injection for the reader. It verifies if the ProVerif response confirms that the connection on the reader is successfully opened and closed. The query result indicates that the event injection from end_R(IDR[]) to start_R(IDR[]) is true, thereby indicating that the reader’s communication channel is functioning correctly.
- Query 3 focuses on the event injection for the tag. It checks if the ProVerif response confirms that the connection on the tag is successfully opened and closed. The query result indicates that the event injection from end_T(IDT[]) to start_T(IDT[]) is true, thus implying that the tag’s communication channel is functioning correctly.
- Query 4 examines the security/strength of the secret key KNEW by checking if it is susceptible to an attacker. The ProVerif response indicates that KNEW is secure, given that the result of not attacker(KNEW[]) is true. Therefore, the secret key KNEW is deemed secure, and an attacker cannot intercept it from the public channel.

The summary of security analysis is provided in Table 3. The four queries in the ProVerif security analysis provide insights into the functionality and security aspects of the system under consideration. By evaluating the ProVerif responses, we can gain confidence in the proper operation of the server, reader, and tag, as well as the security of the secret key.

### 5.2. BAN Logic Security Proof

The accuracy of the designed protocol was checked through BAN logic. The BAN logic notations are shown in Table 4.

- Goal 1: S|≡R≡{Cro(RID⊕TID),K}
- Goal 2: $S|\equiv R|\equiv \left\{Cro\left(RID⊕TID,K⊕N_{S}^{{}^{\prime }}\right)\right\},{\left\{Rot\left(K⊕TID,K⊕RID\right)∥N_{S}^{{}^{\prime }}⊕K\right\}}_{K}$
- Goal 3: $R|\equiv T\left\{Cro\left(RID⊕TID.K_{new}\right)\right\}K_{new}$
- Goal 4: $T|\equiv R\equiv \left\{K_{new}⊕N_{T}^{{}^{\prime }}⊕N_{R}^{{}^{\prime }}\right\}K_{new}$
- Goal 5: $T|\equiv R|\equiv S\overset{K_{new}}{⟷}T$

#### 5.2.1. Idealized Form

Part 1: In the proposed protocol, the idealized form is discussed below:

- M1: $N_{R}^{{}^{\prime }}<R_{R}>KRT$
- M2: $Cro<RID⊕TID>K,NT^{{}^{\prime }}<R_{T}>KRT$
- M3: $Cro<RID⊕TID>K,NR^{{}^{\prime \prime }}<R_{R}>KSR,N_{T}^{{}^{\prime \prime }}<R_{T}>K^{SR}$
- M4: $Cro<RID⊕TID,K⊕NS^{{}^{\prime \prime }}>,Rot<K⊕TID,K⊕RID>,<K⊕N_{S}^{{}^{\prime }}>$
- M5: $<TID>RR,N_{S}^{{}^{\prime \prime }}<R_{S}>KRT$
- M6: $Cro<RID⊕TID,K_{new}<R_{R}⊕R_{S}⊕R_{T},K>>$
- M7: $\left(K_{new⊕R_{T}⊕R_{R}}\right)$
- M8: $\left(Mark⊕R_{S}\right)$

#### 5.2.2. Assumption

Part 2: The following assumptions were made to analyze the designed scheme using BAN logic.

- A 1: $T|\equiv \;T\overset{K}{⟷}R,R|\equiv \;R\overset{K}{⟷}T$
- A 2: $R|\equiv \;R\overset{K}{⟷}S,S|\equiv \;S\overset{K}{⟷}R$
- A 3: $S|\equiv \;S\overset{K}{⟷}T,T|\equiv \;T\overset{K}{⟷}S$
- A 4: $R|\Rightarrow R_{R},R|\equiv \#\left(R_{R}\right),R\left|\equiv T\right|\equiv S\overset{R_{R}}{\Rightarrow }R$
- A 5: $T|\Rightarrow R_{T},T|\equiv \#\left(R_{T}\right),T\left|\equiv R\right|\equiv S\overset{R_{T}}{\Rightarrow }T$
- A 6: $S|\Rightarrow R_{S},S|\equiv \#\left(R_{S}\right),S\left|\equiv R\right|\equiv T\overset{R_{S}}{\Rightarrow }S$
- A 7: T|≡R≡S≡#(K)

#### 5.2.3. Idealized form Verification

Part 3: With the goals and idealized form set up, the proposed scheme can be verified using BAN logic.

Through the use of the Q⊲X seeing rule,

- V 1: $S<{Cro(RID⊕TID,K)}_{K},N_{R}^{{}^{\prime }},N_{T}^{{}^{\prime }}\left(A2\right)$, which demonstrates that only the reader and the server (as well as any other entities that they believe know the value of K) can access S. Combining this with the message seeing rule, P < (X, Y) |- P < X, we obtain

- V 2: $S{\left\{Cro\left(RID⊕TID,K\right)\right\}}_{K}$, where Cro is a cryptographic function, RID and TID are identifiers, and *K* is the shared secret key.

According to line V 2 and the msg-meaning rule, which is $\frac{Q|\equiv Q\overset{K}{⟷}T.q⊲<X{>}_{K}}{Q|\equiv T|∼X}$, we attain

- V 3: $S\equiv R|\neg {\mathrm{Cro}(RID⊕TID,K)}_{K}$

Using the rule of Freshness $\frac{Q|\equiv \#(X)}{Q|\equiv \#(X,Y)}$ and V 3, we attain

- V 4: $S\equiv S⊕{\left\{\mathrm{Cro}\left(RID⊕TID,K\right)\right\}}_{K}$

By the use of the nonce verification $\frac{Q|\equiv \#(X),Q|\equiv T|∼X}{Q|\equiv T|\equiv X}$ rule, we attain

- V 5: $S\equiv R\equiv {\mathrm{Cro}(RID⊕TID,K)}_{K}$
  Hence, according to the above proof process, the first goal (Goal 1) has been achieved. Similarly, we can compute the message sent to the reader from the server as
- V 6: $R<\left\{Cro\left(RID⊕TID,K⊕N_{S}^{{}^{\prime }}\right).Rot\left(K⊕TID,K⊕RID\right)N_{S}^{{}^{\prime }}⊕K\right\}{>}_{K}$, namely, the Goal 2.
  By the same procedure, we can compute Goals 3 and 4. According to (A 1, A 2, A 3) and the process of front demonstration, we can obtain $T\equiv R^{K}\overset{K_{\mathrm{new}}}{⟷}T,\;\mathrm{and}\;R\equiv S\overset{K_{\mathrm{new}}}{⟷}R$. Moreover, we combine secret rules and message keys. Given $P\equiv R\overset{K}{⟷}R_{1}\equiv P\equiv R_{1}\overset{K}{⟷}R$ and $P\equiv Q\equiv R\overset{K}{⟷}R_{1}\equiv P\equiv R_{1}\overset{K}{⟷}R$, we can see that
- V 7: $T\equiv_{R}S\overset{K_{\mathrm{new}}}{⟷}T$
  Hence, all the protocol goals have been proved to secure the proposed scheme logically.

#### 5.2.4. Goals

There are two participators—the authorized user ($U_{i}$) and the authorized server ($LS_{j}$)—in our proposed protocol. Four goals were set to satisfy the correctness of the designed authentication scheme.

1. The server $LS_{j}$ believes that $U_{i}$ and $LS_{j}$ share a secret parameter $DID_{i}$;
2. $LS_{i}$ believes in $U_{i}$ and $U_{i}$ also believes that $U_{i}$ and $LS_{j}$ share the secret value $DID_{i}$;
3. $U_{i}$ believes that $LS_{j}$ shares the secret key of $DID_{i}$ with $U_{i}$;
4. $U_{i}$ believes in $LS_{j}$ and also believes that $LS_{j}$ shares a secret key $DID_{i}$ with $U_{i}$.

These four goals in the language of the BAN logic are exposed as Goal-1 and Goal-2. BAN logic has proved that $U_{i}$ and $LS_{j}$ attain mutual authentication and securely achieve the session key agreement. Consequently, it can be concluded that the proposed authentication scheme is correct.

## 6. Informal Security Analysis

In the previous section, a formal analysis of the proposed security scheme was conducted using well-known automated tools such as ProVerif and BAN Logics, thus validating its correctness. Building upon the formal analysis, this section focuses on an informal security analysis, which compares the proposed scheme with existing protocols to meet various security criteria, as shown in Table 5.

The informal security analysis involved a comparison of the proposed scheme with the Chien Protocol [10], Gossamer Protocol [11], Xie Protocol [13], Sarah Protocol [12], and Fan Protocol [14]. Table 6 presents the results of this comparison, which showcase how the proposed scheme fared against each protocol in fulfilling the listed security standards.

Upon examining Table 6, it becomes apparent that the proposed scheme outperformed all the compared protocols in meeting the specified security criteria. It achieved a score of one (provides) for all security criteria (R1–R9), thus indicating its capability to fulfil all the requirements. In contrast, the other protocols exhibited varying degrees of effectiveness in meeting the security criteria.

Based on the comparison, it is evident that the proposed scheme exceled in fulfilling all the listed security criteria (R1–R9). It effectively provided tag anonymity, protected against reply and synchronization attacks, ensured forward secrecy, as well as mutual authentication, and guarded against DoS attacks, impersonation attacks, insider attackers, and formal verification. These findings reinforce the robustness and effectiveness of the proposed security scheme, as validated by both the formal analysis and the informal comparison.

Considering the formal analysis results and the strengths highlighted in the informal comparison, it can be concluded that the proposed security scheme offers a robust and comprehensive solution to meet security requirements when compared to the existing protocols.

## 7. Conclusions

We presented a lightweight RFID protocol that effectively addresses existing schemes’ anonymity and traceability issues. Using pseudonyms instead of real IDs, our proposed protocol ensured patient privacy while establishing secure communication between tags and readers. The protocol has undergone rigorous testing and has demonstrated resilience against various security attacks. We firmly believe that our proposed protocol can contribute to developing secure and privacy-preserving healthcare systems in the context of the Internet of Things.

As part of our future work, we plan to conduct comprehensive simulations to evaluate the proposed protocol under realistic conditions. These simulations will enable us to assess the protocol’s performance metrics in various deployment scenarios, such as communication latency, scalability, and resource utilization. These simulations aim to bridge the gap between theoretical analysis and real-world applicability, thereby providing concrete evidence of the protocol’s effectiveness and efficiency.

## Figures and Tables

**Table 1:** Notations used in formal representation of proposed authentication scheme.

Notations			
**Notation**	**Description**	**Notation**	**Description**
$R_{R}$	Random Nonce of Reader	$R_{S}$	Random Number of Server
$R_{T}$	Random Number of Tag	TID	Tag ID
RID	Reader ID	$K_{RT}$	Preshared Key between Reader and Tag
$K_{SR}$	Preshared Key between Server and Reader	⊕	The XOR Operation
*K*	Current Session Number	$K_{new}$	Next Session Number
Cro(x,y)	Cross Operation	Rot(x,y)	Rotation Operation, x=W(y)
Mark	Status of Last Session	$N_{R}^{{}^{\prime }}$	Random number of Reader $R_{R}$ xor with $K_{RT}$
$N_{T}^{{}^{\prime }}$	Random number of Tag $R_{T}$ xor with $K_{RT}$	$N_{S}^{{}^{\prime }}$	Random number of server $R_{S}$ xor with $K_{SR}$
$N_{T}^{{}^{\prime \prime }}$	Random number of Tag $R_{T}$ xor with $K_{SR}$	$N_{S}^{{}^{\prime \prime }}$	Random number of server $R_{S}$ xor with $K_{RT}$

**Table 2:** Computation cost comparison (∧ represents exponentiation, ⊕ indicates the XOR operation, “||” is the cascading operation, “Hash” is the hash operation, and “Cro” is the cross operation defined previously. Similarly, PRNG stands for pseudo-random number generator, while “Rot” indicates the displacement operation, and the cost of operations such as ⊕ and “Rot” are relatively lower).

Schemes	Cost
Kaul et al. [15].	⊕,PRNG,Hash
Chien Protocol [10].	⊕,∧,||,Rot
Gossamer Protocol [11].	$⊕,\wedge ,Rot^{2}$
Xie Protocol [13].	⊕,||,Hash
Sarah Protocol [12].	⊕,∧,||,Hash
Fan Protocol [14].	⊕,||,Cro,Rot
Proposed Scheme	⊕,Cro

**Table 3:** ProVerif security analysis.

Query	ProVerif Response
Query inj-event(end_S(IDS[])) ==>inj-event(start_S(IDS[])) Completing… Starting query inj-event(end_S(IDS[])) ==>inj-event(start_S(IDS[]))	inj-event(end_S(IDS[])) ==>inj-event(start_S(IDS[])) is true.
Query inj-event(end_R(IDR[])) ==>inj-event(start_R(IDR[])) Completing… Starting query inj-event(end_R(IDR[])) ==>inj-event(start_R(IDR[]))	inj-event(end_S(IDR[])) ==>inj-event(start_S(IDR[])) is true.
Query inj-event(end_T(IDT[])) ==>inj-event(start_T(IDT[])) Completing… Starting query inj-event(end_T(IDT[])) ==>inj-event(start_T(IDT[]))	inj-event(end_T(IDT[])) ==>inj-event(start_T(IDT[])) is true.
Query not attacker(KNEW[]) Completing… Starting query not attacker(KNEW[])	not attacker(KNEW[]) is true.

**Table 4:** Notations table for BAN logic.

Notations	Description
Q|≡X	Q believing in X
Q⊲X	Q sees which is X
Q|≡T	Q believes T’s action. E.g., Q|≡T|≡X
	means Q believes T believes X is true
Q|∼X	Q once says X
Q⇒X	Q has full jurisdiction beyond X
#(X)	X is updated and fresh
${\left(C\right)}_{k}$	Combine conditions C by the use of k
${\left(C\right)}_{k}$	Carry out hash operation on C; use X
${\left(X\right)}_{K}$	Message of hash X with a key K
$Q\overset{k}{⟷}T$	Q and T used to interact using the shared key k
	with each other
$DID_{i}$	Session key $DID_{i}$ used one time in the
	current section
$\frac{Q|\equiv Q\overset{K}{⟷}T.q⊲<X{>}_{K}}{Q|\equiv T|∼X}$	Rule of Message-Meaning
$\frac{Q|\equiv \#(X)}{Q|\equiv \#(X,Y)}$	Rule of Concatenation-Freshness
$\frac{Q|\equiv \#(X),Q|\equiv T|∼X}{Q|\equiv T|\equiv X}$	Rule of Verification-Nonce
$\frac{Q|\equiv T\Rightarrow X,Q|\equiv T|\equiv X}{Q|\equiv X}$	Rule of Jurisdiction

**Table 5:** List of security criteria.

Security Criteria	Description
Tag Anonymity (R1)	Tag anonymity ensures privacy and prevents unauthorized tracking by concealing the identity of the tag or device that transmits information in a system or protocol.
Reply Attack (R2)	A malicious actor intercepts and retransmit legitimate data or actions to deceive a system, thereby compromising its integrity and security.
Synchronization Attack (R3)	It occurs when an attacker manipulates the coordination among the different entities to disrupt normal operations or gain unauthorized access. This attack compromises the targeted system’s integrity, availability, or confidentiality by exploiting timing or communication dependencies.
Forward Secrecy (R4)	A security vulnerability where the exposure of a long-term secret key does not compromise the privacy of previous communications. This ensures that historical data remains safeguarded, even if the private key is compromised.
Mutual Authentication (R5)	Mutual authentication is a security measure where both parties involved in a communication process verify each other’s identities, thereby establishing trust and preventing unauthorized access or impersonation. This ensures that the reader, tag and server confirm each other’s authenticity before establishing a connection.
DoS Attack (R6)	An adversary inundates a target system or network with high requests or traffic, thus resulting in service disruption or unavailability for legitimate users. The goal is to deplete system resources and impede its ability to handle legitimate requests.
Impersonation Attack (R7)	It occurs when an attacker assumes a false identity by posing as a legitimate user or entity in a cybersecurity breach. By exploiting this deception, the attacker aims to gain unauthorized access, deceive others, and potentially engage in malicious actions such as manipulating or stealing sensitive information while bypassing security measures.
Insider Attacker (R8)	It occurs within an organization and involves trusted individuals such as employees or contractors with authorized access. Leveraging their privileged positions, these attacks target system compromises, data theft, or infrastructure damage, thus posing significant risks due to the insider’s knowledge and authorized access.
Formal Verification (R9)	Formal verification means the proposed scheme security test uses well-known automated tools such as ProVerif. It also test the correctness of the proposed scheme using BAN Logics.

**Table 6:** Informal security proof table. 1: provides, 0: Does not provide.

Schemes	R1	R2	R3	R4	R5	R6	R7	R8	R9
Chien Protocol [10].	0	1	1	0	1	0	0	0	0
Gossamer Protocol [11].	1	0	0	1	1	0	0	0	0
Xie Protocol [13].	0	1	1	1	0	1	0	0	0
Sarah Protocol [12].	0	1	1	1	1	1	0	0	0
Fan Protocol [14].	0	1	1	0	1	0	0	0	0
Proposed Scheme	1	1	1	1	1	1	1	1	1

## Data Availability

Available upon request.
